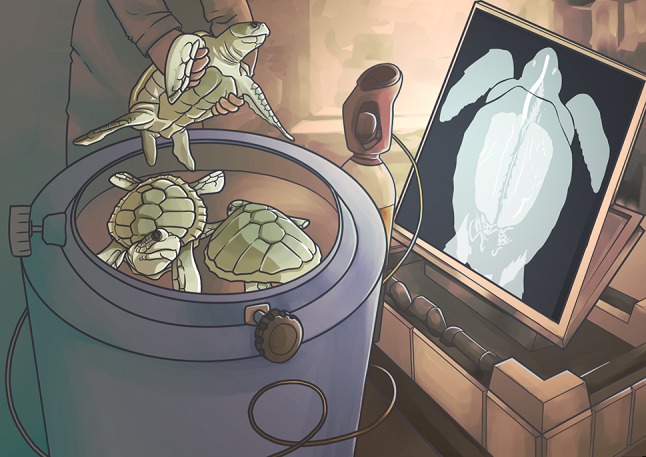# Sea turtles with “the bends” breathe easy again after oxygen therapy

**DOI:** 10.1093/conphys/coy027

**Published:** 2018-06-08

**Authors:** Christine L Madliger

**Affiliations:** Department of Biology, Carleton University, 1125 Colonel By Dr., Ottawa, Ontario, Canada

Loggerhead sea turtles sometimes end up accidentally entangled in fishing nets. With the knowledge that many turtles in this situation are suffering respiratory distress that could be fatal, a team of researchers and veterinarians in Spain have made it their mission to find a way to improve turtle survival rates.

Decompression sickness, or “the bends”, occurs when gas bubbles form in the blood and other tissues like the lungs. In humans, decompression sickness is most common in scuba divers who ascend too quickly to the surface after a deep dive. But this illness can also arise in marine animals, like sea turtles, when they surface following unnaturally intense exercise or stress, like that which occurs during entrapment in fishing gear. As a result, sea turtles suffer from reduced blood flow to their lungs and difficulty breathing, which can ultimately lead to their death.

Luckily, sea turtles that are rescued following entanglement in fishing gear now have a much greater chance of recovering due to oxygen treatment using a specialized hyperbaric (pressurized) chamber. In a recent study, Cyril Portugues and his colleagues (2018) sought to measure lung function in turtles with decompression sickness prior to and after oxygen therapy. They hoped to gauge how well the turtles were recovering. When turtles were admitted to the rehabilitation centre, the researchers quantified the severity and position of gas bubbles, also called emboli, using radiography and ultrasounds. They then measured each turtle’s lung function by placing a small mask over the turtle’s face that could record how quickly the animal was breathing, the duration of each breath, and the amount of air flowing in and out of the lungs. Turtles were then placed in the oxygen chamber for 12–14 h after which they were removed and their lung function was reassessed both immediately and then again weekly until the animals could safely be released back into the wild.

Portugues and his team found that the oxygen treatment immediately increased respiratory flow by up to 45% and the amount of air that the sea turtles could take in during each breath by up to 35%. In other words, the turtles could breathe much more comfortably! Overall, the team has produced strong evidence demonstrating that loggerhead sea turtles suffering from decompression sickness have impaired respiratory function. But, this can be greatly improved with hyperbaric oxygen treatment.

In Valencia, Spain, where this research was conducted, collaboration between local government and fishers has allowed turtle–fisher interactions to be better quantified and reported. Pairing this type of on-the-ground information with physiological measurements will not only improve rehabilitation programs for injured turtles, but could help determine ways to diminish turtle bycatch by fisheries in the first place.

Illustration by Erin Walsh; Email: ewalsh.sci@gmail.com